# Determining the societal value of a prospective drug for ME/CFS in Germany

**DOI:** 10.1371/journal.pone.0307086

**Published:** 2024-07-18

**Authors:** Afschin Gandjour

**Affiliations:** Frankfurt School of Finance & Management, Frankfurt, Germany; Shiraz University of Medical Sciences, ISLAMIC REPUBLIC OF IRAN

## Abstract

**Background:**

Myalgic Encephalomyelitis/Chronic Fatigue Syndrome (ME/CFS) imposes a substantial societal and economic burden. The aim of this study is to ascertain the optimal level of public research and development (R&D) investment in Germany for a prospective drug, given the pressing need for effective treatments.

**Methods:**

This study calculates the societal value from a German perspective by integrating health and economic outcomes in the context of public R&D investment for ME/CFS. It considers factors such as direct medical costs, productivity loss, and the effectiveness of a prospective drug.

**Results:**

The anticipated introduction of a prospective drug is estimated to yield a quality-adjusted life year (QALY) gain of approximately 29,000 and a societal value of about €2.6 billion. The optimal R&D investment in Germany is estimated at €676 million, which represents about a quarter of the total investment required to bring a significant drug to market, considering diminishing returns and market constraints. Results were confirmed in the sensitivity analysis.

**Conclusions:**

The study concludes that a coordinated international approach is imperative to address the funding and market size limitations effectively in developing treatments for ME/CFS and to realize the substantial societal and economic benefits.

## Introduction

Myalgic Encephalomyelitis/Chronic Fatigue Syndrome (ME/CFS) is a severe chronic condition profoundly impairing quality of life. The health-related quality of life for those with ME/CFS is notably lower than the average population and is worse than that observed in 20 other conditions, including lung cancer and multiple sclerosis [1]. The condition is characterized by extreme exhaustion, pain, sleep disturbances, and concentration problems. These symptoms can intensify for days or weeks following minimal physical or mental activities. This sensitivity to exertion, referred to as post-exertional malaise (PEM), is a defining feature of ME/CFS [2].

Currently, no medication has received approval for ME/CFS. Treatment for ME/CFS typically involves managing symptoms through activity pacing, sleep improvement, pain management, mental health support, and lifestyle adjustments, with an individualized approach to address the specific needs of each patient, but there is no known cure [3]. The pursuit of alleviating suffering juxtaposed with the necessity for stringent testing of interventions presents a significant dilemma in this field (cf. [4]). Several factors contribute to this lack of approved treatments:

Firstly, the diverse range of symptoms and the lack of objective criteria obstruct the process of accurately diagnosing ME/CFS [5]. The absence of definitive biomarkers exacerbates the complexity of diagnosis and management, leaving a substantial number of individuals undiagnosed [6]. This hampers the identification of patient subgroups that might benefit from specific therapies.

Diagnostic data indicate that the prevalence of ME/CFS in Germany ranged between 350,000 and 400,000 during the pre-pandemic years of 2018 and 2019, with a surge to nearly 500,000 patients nationwide observed in 2021 [7].

Secondly, the progression of the illness is highly variable, with symptoms fluctuating over days, weeks, or even longer, and some individuals may undergo periods of remission [8, 9]. Outcomes can range from complete recovery to partial improvement or even worsening.

Thirdly, research into ME/CFS is notably lagging compared to diseases of similar disease burden [10]. The dearth of research into the disease mechanisms and diagnosis of ME/CFS, coupled with prevailing misconceptions about its nature—such as perceptions of it being a psychological or psychosomatic illness—has likely hindered early investment in research.

Fourthly, the scant state-funded research is partly due to the free-rider phenomenon, where countries depend on each other’s research outputs, resulting in state failure and gaps in scientific understanding [11].

Fifthly, due to the subtle nature of ME/CFS, which often involves no visible physical changes and has low mortality rates, there is a risk that physicians may underestimate the severity of the disease and harbor negative attitudes [12], potentially affecting the prescription of medications that might become available in the future.

Despite ME/CFS’s relatively high prevalence, the aforementioned issues have led to a dearth of investment by the pharmaceutical industry in research and development (R&D) for ME/CFS, especially in clinical trials, resulting in market failure. Such failure would typically prompt state support for basic and clinical research.

To tackle these challenges, a potential strategy could involve testing existing drugs for other indications. However, given the limited incentives for pharmaceutical companies, especially when generics or biosimilars exist, state intervention may be indispensable. The free-rider phenomenon implies that coordinated global or, minimally, European research funding may be essential, with an international alliance offering success-based incentives potentially accelerating the development of new medications.

While there has been a call for expansive trials on ME/CFS [13], smaller, intensive therapy-focused proof-of-concept studies could hone in on biological measurements in a select group to elucidate the role of specific mechanistic pathways. Larger, randomized trials could assess the efficacy of scalable interventions on symptom outcomes in extensive cohorts. Both niche and extensive approaches, and variations therein, potentially hold value (cf. [4]).

The pharmaceutical industry’s investment decisions gravitate towards the more lucrative markets, predominantly the United States. Hence, awareness campaigns isolated to Germany would likely fall short. A more targeted campaign in the United States could potentially amplify awareness and advance R&D of treatments.

Importantly, even with understood disease mechanisms and significant market sizes, navigating a drug to market is fraught with challenges. For instance, Alzheimer’s drug development, despite its understood pathogenesis and substantial market, faces a staggering 99.6% failure rate, the highest among drug research sectors [14]. Promising drugs like donanemab and lecanemab only mildly ameliorate cognitive decline and present serious concerns like brain bleeding and swelling [15].

Additionally, the competition for private and public funding for ME/CFS is stiff, not only with other diseases but also with other public sectors like infrastructure and environmental protection.

This study aims to discern the optimal public R&D investment in Germany for developing a new ME/CFS drug. Public investment, especially in the context of ME/CFS in Germany, is primarily aimed at serving the national population’s health needs. In contrast, pharmaceutical companies often pursue R&D with a global market in view, driven by potential profitability across different regions.

## Methods

### Calculation of the societal value (SV)

This study takes a German societal perspective to determine the optimal level of public R&D investment in ME/CFS by calculating the SV. SV is computed as the sum of monetary gains from health improvements and savings achieved by averting direct medical costs and productivity losses. The methodology involves defining a health improvement value per patient using the minimally important difference (MID), then translating this into monetary terms based on the maximum willingness to pay. This value is multiplied across the patient population, factoring in the discount rate over time to obtain the SV for health gains:

$$SV_{\text{health}}=MID\cdot \lambda \cdot \sum_{t=1}^{T}\frac{n_{t}}{{\left(1+r\right)}^{t}}.$$ (1)

Here, *MID* is the minimally important difference; *λ* stands for the maximum willingness to pay for a health outcome; *n* symbolizes the number of patients in the target population; *t* is the time horizon over which SV is computed; and *r* denotes the discount rate. The period *t* reflects the prolonged effects of ME/CFS on patients and the healthcare system, acknowledging the disease’s chronic nature. The inclusion of a discount rate incorporates the time value of money and health outcomes into the SV calculation.

To estimate the cost savings from lowering direct medical expenses (excluding rehabilitation costs) across the patient group, the direct medical cost per patient is multiplied by the patient population size. Rehabilitation expenses are multiplied by the number of new cases (incidence), considering these costs occur as a one-time event rather than annually. Additionally, the MID is factored into the calculation of direct medical and rehabilitation costs, suggesting a direct correlation between spending on these services and the clinical impact of ME/CFS.


$$SV_{\text{direct}}=MID\cdot \left(c_{\text{direct}}\cdot \sum_{t=1}^{T}\frac{n_{t}}{{\left(1+r\right)}^{t}}+c_{\text{reha}}\cdot \sum_{t=1}^{T}\frac{I_{t}}{{\left(1+r\right)}^{t}}\right).$$ (2)

Here, *c*_direct_ and *c*_reha_ refers to the per-patient costs for direct medical care and rehabilitation, respectively, and *I*_*t*_ represents the number of new cases (incidence rate) in year *t*.

The savings from mitigating indirect costs (i.e., productivity losses) are calculated by multiplying the national average income of employees by the MID. This MID integration into the calculation of productivity losses suggests a proportional relationship between productivity losses and the clinical severity of ME/CFS. This figure is then multiplied by the incidence rather than the prevalence of the patient population, as the latter includes long-term absentees who have already been replaced and therefore do not contribute to productivity losses.


$$SV_{\text{indirect}}=MID\cdot c_{\text{indirect}}\cdot \sum_{t=1}^{T}\frac{I_{t}}{{\left(1+r\right)}^{t}}.$$ (3)

Here, *c*_indirect_ represents the indirect cost per patient.

The aggregation of these three components of societal value results in the total societal value, given by the equation:

$$SV=MID\cdot \left[\left(\lambda +c_{\text{direct}}\right)\cdot \sum_{t=1}^{T}\frac{n_{t}}{{\left(1+r\right)}^{t}}+\left(c_{\text{reha}}+c_{\text{indirect}}\right)\cdot \sum_{t=1}^{T}\frac{I_{t}}{{\left(1+r\right)}^{t}}\right].$$ (4)

As a measure of health outcome, the study utilized quality-adjusted life-years (QALYs). QALYs are a standardized metric that enable comparisons across various medical interventions for different diseases. They combine both the length of life and the quality of life by quantifying individual preferences for various health states, with values anchored on a scale from 0 (representing death) to 1.0 (indicating perfect health). This scale accounts for health states that can have negative values, representing conditions worse than death.

### Data

Table 1 reports the input values and distributions used in the base case and sensitivity analyses. Even with a projected prevalence of 500,000 patients in Germany [7], the considerable disease heterogeneity likely narrows down the number of eligible patients (n) for a future ME/CFS drug due to disparate treatment effects across subgroups. It is improbable for a single drug to benefit all patients. In absence of a ME/CFS-specific heterogeneity measure, an average measure across all diseases was applied. Specifically, data on non-orphan drugs that underwent the German early benefit assessment of new drugs (Arzneimittelmarkt-Neuordnungsgesetz, translated as the Pharmaceuticals Market Reorganisation Act) were employed, considering the average uptake of new drugs in Germany.

**Table 1:** Input values and distributions used in the base case and sensitivity analysis.

Input	Mean (range)	Reference
Clinical and epidemiological data		
ME/CFS prevalence	500,000	[7]
ME/CFS incidence per year	15/100,000	[16]
Average disease population	1,322,051	[17]
Average target population	375,331	[17]
Drug uptake 4 years after launch	17% (10% - 20%)	[18]
Minimally important difference	15%	[19]
Medication adherence in the real world	50%	[20]
Medication adherence in clinical trials	85% (80% - 90%)	[20]
Time horizon (prescription after launch until loss of regulatory data protection)	15 years	[21]
Probability of added benefit by FJC	53%	[17]
Economic data		
National employment rate	76.9%	[22]
Average national employee income per year	€46,700	[23]
Vacancy period	3 months	[24]
Training period for a replacement worker	2 weeks	[25]
Probability of a vacant position	3.7%	[26]
Direct medical costs (without rehabilitation)	€0 (€0 - €5000)	[27]
Costs per rehabilitation stay	€3210	[28, 29]
Work disability rate	60%	[30]

ME, Myalgic encephalomyelitis; CFS, chronic fatigue syndrome; FJC, Federal Joint Committee (Gemeinsamer Bundesausschuss or G-BA)

The analysis also factored in the gradual incorporation of new drugs into clinical practice in Germany, attributed to patient education, treatment engagement, therapy preferences, costs of new medicines, reimbursement and formulary conditions, and physician guidelines (cf. [31]). Given the likely biases against ME/CFS by physicians and anticipated low prescription rates, the 17% uptake at year 4 post-market launch seems realistic, with a presumption of continuous prescription [18].

The health-related quality of life in ME/CFS was evaluated using a self-administered EQ-5D-3L questionnaire, applying Danish population preference weights [1]. The sample included 112 members of the Danish ME/CFS association in 2013/2014, reflecting a response rate of 35% [1].

The minimally important difference (MID) was posited as 15% of the range of respective morbidity or quality of life scales, aligned with the methods delineated by the Institute for Quality and Efficiency in Health Care [19] in Germany. Long-term efficacy of the drug was presumed.

Given the chronic nature of ME/CFS, characterized by fluctuations and remission periods, long-term therapy is imperative for most patients. The SV’s time horizon is influenced by multiple factors including the launch time of an in-class substitute therapy. A 15-year time horizon was assumed, referencing the average uptake of new drugs and prescription volume alterations in Germany [21].

The upper limit of willingness to pay for a health outcome was derived from health system opportunity costs, with a threshold value of €88,107 per QALY gained [32].

To assess productivity loss, the study employed the friction cost method, which is based on the concept of replacing long-term absentees. This method assumes that societal-level production losses are limited [33]. Central to this method is the ‘friction period’—the time interval between when an employee’s position becomes vacant due to ME/CFS-related absenteeism and when that vacancy is filled. In March 2022, over 52% of vacancies in Germany were filled in less than three months [24]. Furthermore, the study incorporated a two-week training period for replacements [25].

While the friction cost approach assumes positions vacated can be filled after a specific period, positions of some patients with long-term or permanent incapacities may not be refilled, necessitating adjustments to the model. In such instances, considering both short-term friction costs and long-term productivity losses in health economic evaluation models is prudent. As of the first quarter of 2023, there were 3.7 vacancies for every 100 positions demanded by companies [26]. For estimating long-term productivity losses, this vacancy rate was projected across the study’s designated time frame.

Precise data regarding direct medical expenditure on ME/CFS in Germany was scarce. Many patients report an extensive, costly journey to receive a conclusive ME/CFS diagnosis [34]. These costs are retrospective estimates and prone to recall bias due to the extensive recall period. Given the divergent treatment patterns and varying physician opinions on causal factors and optimal care, applying guideline-based treatment recommendations to calculate treatment expenditure was debatable. Considering these challenges, the assessment of direct medical costs (excluding rehabilitation expenses) for this condition relied on data from an external source, specifically a study conducted in Australia [27], for the purposes of sensitivity analysis.

However, in the base case, rehabilitation costs were estimated within the assumption that all incapacitated ME/CFS patients would undergo rehabilitation to avert disability. While this assumption may lead to an overestimation of rehabilitation costs, it is also recognized that some patients might participate in rehabilitation multiple times. Due to the lack of specific cost data for ME/CFS rehabilitation stays, estimates were adapted from similar expenses reported for Long Covid patients [28], considering the clinical similarities between the two conditions. Additionally, a societal perspective-based copayment of €10 per day was added [35]. The duration was assumed to be three weeks.

### Diminishing returns of research

The analysis, thus far, presumes a constant return on R&D investment, yielding a consistent QALY gain per euro invested. Nonetheless, research projects exhibit heterogeneous output levels. Based on the diminishing returns principle—each additional investment unit yields progressively smaller gains—it is posited that average R&D investment returns exceed the marginal returns for the last investment unit. This concept is supported by data from Deloitte [36], which illustrates a marked increase in average R&D expenditure per drug launch among ’12 large cap biopharma companies’ over a specified period, with spending rising from $1.188 billion in 2010 to $2.168 billion in 2018. Consequently, an investment yielding a marginal return equivalent to the average return at 100% investment is typically optimal at less than 100% investment, suggesting partial, rather than full, R&D investment is often ideal. Given the fluctuations in the data from Deloitte [36], a square root approximation is employed for simplification (refer to S1 Appendix).

### Comparison with research spending

Recent analyses reveal that investments by the U.S. National Institutes of Health in drugs approved between 2010–2019 are comparable to those of the pharmaceutical industry when accounting for basic and applied research, failed trials, and capital costs or discount rates [37], approximating $3 billion per approval. This expenditure is primarily driven by basic research costs. Applying a 3% discount rate, deemed consistent with prevailing economic principles, the estimate reduces to approximately $1.8 billion per approval [37]. However, these calculations do not consider that, in the German market, merely 53% of new non-orphan drugs have demonstrated added benefit over standard care [17]. Contributory factors include a lack of clinical significance or the achievement of the MID of 15% of the respective scales as stipulated by IQWiG. When the lack of added benefit in numerous new drugs is accounted for, government research spending escalates to roughly €3.3 billion per approval.

### Sensitivity analysis

One-way deterministic analyses were conducted to assess parameter uncertainty by individually varying each input parameter that was subject to variation.

## Results

The anticipated QALY gain from a prospective drug in the German population is projected to be around 29,000 over the relevant lifetime, translating to a monetary value of approximately €2.5 billion, based on the German opportunity-cost threshold. The alleviation of productivity loss and the reduction in direct medical costs due to ME/CFS by a prospective drug are approximated to be about €142 million and €13 million, respectively, over the relevant lifetime. Consequently, the total societal value derived from these benefits is estimated to be €2.7 billion.

Employing a simplified square root approximation reveals that the research investment to achieve a marginal return equivalent to the average return at 100% investment is about 25% of the total, or roughly €676 million. This implies that an R&D investment of roughly 18% of the total costs by the German government would be justified to bring a new drug with a 15% improvement in health outcomes to the market.

### Sensitivity analysis

**Fig 1:**
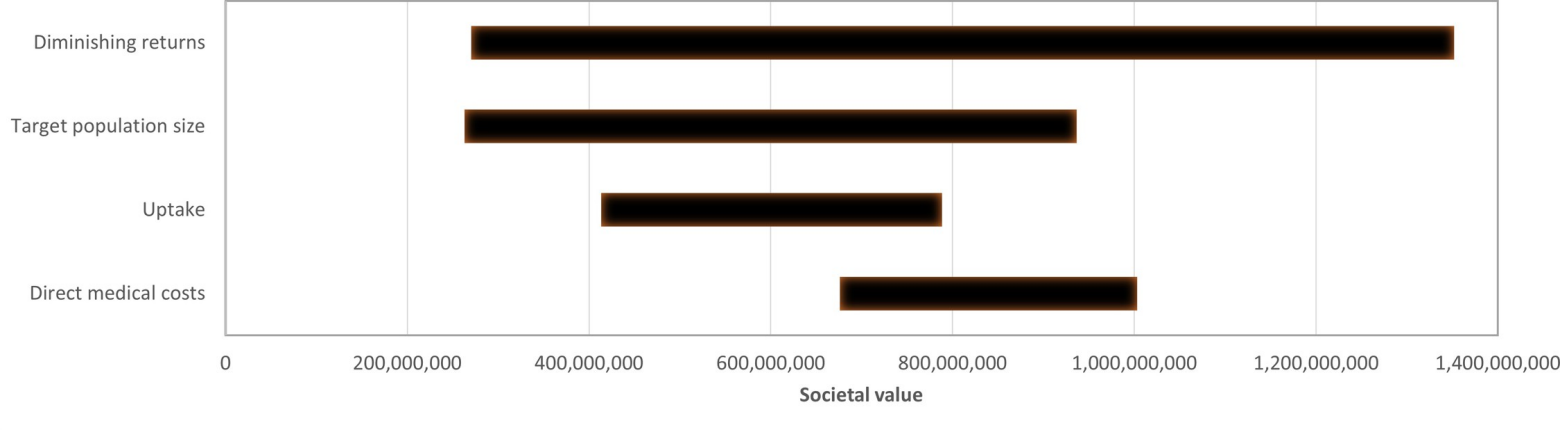
Tornado diagram demonstrating the results of the one-way sensitivity analysis. The variables are ordered by the societal value of drug research and development (in euros).

The outcomes of the one-way sensitivity analysis are illustrated in the tornado diagram in Fig 1. The factor with the most significant influence on the societal value of investing in drug R&D for ME/CFS in Germany is the extent of the diminishing returns of research.

## Discussion

This study illustrates that a prospective drug for ME/CFS holds substantial value predominantly in mitigating clinical burden, exceeding its impact on reducing both productivity loss and direct medical costs. It further elucidates that, from a societal standpoint in Germany, the optimal public investment in basic and applied research for a novel ME/CFS drug is less than what is presumed necessary to bring a significant drug to the market. This discrepancy arises due to the diminishing returns of R&D investment coupled with the constraints of the German market size, a finding corroborated by sensitivity analysis. Therefore, a pragmatic approach would entail seeking international collaborations to secure sufficient investment, emphasizing the imperative for either global or, minimally, European research funding. The success of global initiatives, such as the Medicines for Malaria Venture, which targets tropical diseases and antibiotic resistance, underscores the effectiveness of collaborative efforts in advancing drug development for underserved conditions [38].

The German Federal Ministry of Education and Research intends to facilitate the emergence of innovative, targeted therapeutic strategies by allocating 15 million euros over three years to research the fundamental disease mechanisms of ME/CFS [39]. Meanwhile, the NIH’s allocation for ME/CFS research has remained static at $13–17 million annually since 2017, with the years 2023 and 2024 projected to continue this trend [40]. This nominal investment—totaling $103 million until 2022—possibly mirrors a deficit in grant proposals or a historical reluctance to fund such endeavors, deterring researchers after experiencing rejection, a situation emblematic of the ‘chicken or the egg’ causality dilemma.

The calculations presented in this study fill a notable gap in the literature and, as such, cannot be directly compared with the findings of other studies. For instance, the estimation of disability-adjusted life years (DALYs) lost to ME/CFS, reflecting the entire clinical burden [10], is significantly broader than the reduction in burden this study calculates could be achieved by a single drug. In a similar vein, while estimates on the total research funding for ME/CFS take into account the comprehensive clinical burden in comparison with other diseases [10], they do not isolate the investment in R&D for developing a single medication.

This study predominantly centered on novel drug development. However, repurposing drugs, already approved for alternate conditions, which address identified mechanisms in ME/CFS, might expedite clinical approval processes. An example of this strategy includes the use of Pyridostigmine, originally used for Postural Orthostatic Tachycardia Syndrome (POTS), which has shown promise in ME/CFS patients by improving exercise capacity [41]. This approach exemplifies the reuse of alternative arbitrator drugs for ME/CFS. Furthermore, adopting diverse treatment approaches—comprising pharmacological treatments, physical therapy, nutritional support, and psychological support—aims to manage symptoms and enhance the quality of life for those affected by this condition. Nonetheless, evidence supporting specific combinations of these approaches, including the strategic use of alternative arbitrators, remains limited.

Willingness to pay was evaluated based on health system opportunity costs, but optimal resource allocation across varied public sectors necessitates the application of a societal willingness-to-pay threshold, which might diverge from health system opportunity costs.

Contrary to the adopted friction cost approach in this study, the more prevalent human capital approach for estimating productivity costs (e.g., [42]) has received criticism for potentially overestimating productivity losses. Conversely, this study did not account for productivity losses amongst caregivers, costs related to ‘presenteeism,’ compensation mechanisms, and multiplier effects, implying that the actual costs are likely higher. Additionally, the cost implications of adverse events were not incorporated into the analysis.

For future research, a comprehensive exploration into the underpinnings of ME/CFS is imperative to develop targeted therapeutic strategies, with emphasis on elucidating disease mechanisms and potential drug repurposing, which may expedite clinical approval compared to developing new drugs. A meticulous economic analysis encompassing overlooked aspects such as productivity losses among carers, ‘presenteeism’, and compensation mechanisms is crucial for a holistic understanding of the economic ramifications of ME/CFS and the value of interventions. Additionally, investigating societal willingness to pay is essential to optimize resource allocation across public sectors, especially when deviating from health system opportunity costs. Lastly, embracing a diverse treatment approach by investigating a spectrum of interventions beyond pharmacological solutions, including lifestyle, nutritional, and psychological strategies, can potentially yield more effective and encompassing solutions for ME/CFS sufferers.

## Supporting information

S1 Appendix Formal analysis of diminishing returns utilizing the square root function. (DOCX)
