# Supplementary material for: Determining the societal value of a prospective drug for ME/CFS in Germany
Source: PLoS One. 2024 Jul 18;19(7):e0307086. doi: 10.1371/journal.pone.0307086 (PMC11257356; doi:10.1371/journal.pone.0307086)
Supplement: S1 Appendix — (DOCX) [file pone.0307086.s001.docx]

Appendix. Formal analysis of diminishing returns utilizing the square root function

Given the square root function $f\left( x \right)=k\sqrt{x}$​, where $k$ is a constant, and the average return at 100% is $\lambda$, we have:

$f\left( 1 \right)=k\sqrt{1}=\lambda$. (A1)

Solving for $k$:

$k=\lambda.$ (A2)

Let us now determine the value of $x$ at which the marginal return is equivalent to $\lambda$. The derivative of the square root function $f\left( x \right)=\lambda\sqrt{x}$​​ with respect to $x$ is:

$f'\left( x \right)=\frac{\lambda}{2\sqrt{x}}$. (A3)

Setting this derivative equal to $\lambda$:

$\frac{\lambda}{2\sqrt{x}}=\lambda$. (A4)

Solving for $x$:

$\frac{1}{2\sqrt{x}}=1$ (A5)

$x=\frac{1}{4}$. (A6)

So, for the square root function $f\left( x \right)=\lambda\sqrt{x}$​, an output value of $\frac{1}{4}$​ (25%) yields a marginal return that corresponds to the average return at 100% ($\lambda$). This means that investing 25% of the maximum input yields a marginal return that matches the average return at 100% of the output.
